# An Atypical Presentation of Pyridoxine-Dependent Epilepsy Diagnosed with Whole Exome Sequencing and Treated with Lysine Restriction and Supplementation with Arginine and Pyridoxine

**DOI:** 10.1155/2022/7138435

**Published:** 2022-08-30

**Authors:** Jiyoung Kim, Angela Pipitone Dempsey, Sun Young Kim, Meral Gunay-Aygun, Hilary J. Vernon

**Affiliations:** McKusick-Nathans Department of Genetic Medicine, Johns Hopkins University School of Medicine, Baltimore, MD, USA

## Abstract

Pyridoxine dependent-developmental and epileptic encephalopathy (PD-DEE) or pyridoxine-dependent epilepsy (PDE) is a rare autosomal recessive disorder caused by biallelic pathogenic variants in ALDH7A1. It classically presents as intractable infantile-onset seizures unresponsive to multiple antiepileptic drugs (AEDs) but with a profound response to large doses of pyridoxine (B6). We report a case of PDE with an atypical clinical presentation. The patient presented at 3 days of life with multifocal seizures, fever, increased work of breathing, decreased left ventricular systolic function, and lactic acidosis, raising suspicion for a mitochondrial disorder or infectious process. Within 1.5 weeks of presentation, seizure activity resolved with antiepileptic therapy. Whole exome sequencing (WES) revealed homozygous pathogenic variants in ALDH7A1 (c.1279G > *C*, p.E427Q) and confirmed the diagnosis of PDE. Follow-up biochemical testing demonstrated elevated urine pipecolic acid. In the second week of life, the patient was initiated on triple therapy, including pyridoxine supplementation, low lysine diet, and arginine supplementation, which he tolerated well. Urine pipecolic acid levels responded accordingly after initiation of therapy. Our case illustrates the diagnostic challenges in PDE, the utility of rapid WES in such cases, and the response in urine pipecolic acid to therapy.

## 1. Introduction

Pyridoxine dependent-developmental and epileptic encephalopathy (PD-DEE) or pyridoxine-dependent epilepsy (PDE, OMIM #266100) is a rare autosomal recessive disorder caused by biallelic pathogenic variants in ALDH7A1 located at the locus 5q23.2 with an estimated incidence of 1 : 64,352 live births [1, 2]. PDE classically presents as intractable infantile-onset seizures unresponsive to multiple antiepileptic drugs (AEDs). PDE was first described by Hunt et al in 1954 [2]. Since then, much has been discovered about the biomolecular pathways involved in PDE and etiology behind pyridoxine treatment for seizures in these individuals.

ALDH7A1 encodes for alpha-aminoadipic semialdehyde dehydrogenase, historically called antiquitin, which catalyzes the conversion of alpha-aminoadipic semialdehyde (AASA) to alpha-aminoadipic acid. A defect in this conversion causes an accumulation of metabolic precursors, which notably include pipecolic acid, delta1-piperideine-6-carboxylate (P6C), and the Schiff base of AASA. P6C undergoes the Knoevenagel condensation reaction with pyridoxal-5-phosphate (PLP), the active metabolite of vitamin B6, which causes secondary depletion of PLP. PLP is an important cofactor in neurotransmitter metabolism, and this secondary depletion is thought to cause seizures, with high doses of pyridoxine treating this deficiency and thus the seizures [4, 5].

In the classic presentation of PDE, infants experience intractable seizures with a profound response to the administration of pyridoxine. Atypical PDE may present as later-onset seizures that begin in late infancy, and initial presentation has been described in a patient as old as 18 [6]. Seizures may initially respond to AEDs and/or may not initially respond to pyridoxine, creating diagnostic challenges [7]. Biochemical and genetic testing aids in diagnosis.

Early diagnosis is important in order to initiate prompt treatment. However, reports have indicated that although early diagnosis leads to control of seizure activity, this does not necessarily correlate with prevention of neurocognitive or intellectual delay [8]. Possible reasons for this may be that irreversible damage has already occurred in utero during brain development, the threshold of pyridoxine depletion at which neuronal damage occurs is lower than the threshold at which seizures occur, or there is another mechanism by which neurocognitive delay occurs.

Consensus guidelines for treatment were published in 2021 with updated guidelines to therapy, consisting of pyridoxine, a low lysine diet and arginine supplementation, or “triple therapy” [9]. These recommendations were created out of an understanding that while pyridoxine treats secondary deficiency of pyridoxine, this does not treat the accumulation of toxic precursor metabolites caused by the inability to fully catabolize lysine. Thus, a low lysine diet restricts the amount of substrate, while the supplementation of arginine decreases the amount of lysine passing through the blood-brain barrier by means of competitive inhibition.

Here, we report a case of a newborn diagnosed with PDE through rapid whole exome sequencing and treated in accordance to recently published consensus guidelines.

## 2. Case Presentation

A 3-day-old male infant was initially presented to the hospital with increased work of breathing, grunting, retractions, and an axillary fever of 104 degrees Fahrenheit. At the hospital, he was noted to have multiple episodes of apnea and desaturation requiring vigorous stimulation and oxygen for recovery. Some of these events were associated with arching and abnormal eye movements, concerning for seizure activity. A sepsis investigation including urine, blood, and cerebrospinal fluid cultures was undertaken, and empiric antibiotic therapy with ampicillin, gentamicin, and acyclovir was initiated. All cultures were negative.

Laboratory testing on day of life 4 demonstrated an elevated cerebrospinal fluid lactate of 4.2 mmol/L (normal range 1.1–2.8) and elevated whole blood lactate of 6.3 mmol/L (normal range 0.5–2.0). Ammonia was normal at 58 mcmol/L. Urine organic acids demonstrated moderately increased lactate and increased 2-hydroxyisovalerate, 4-hydroxyphenyllactate, and 4-hydroxyphenylpyruvate. An acylcarnitine profile was normal, though free carnitine and total carnitine were low at 7 *μ*mol/L (normal range for age 10–21) and 13.8 *μ*M (normal range 17–41), respectively. Plasma amino acids demonstrated low citrulline at 5 *μ*M (10–34) and mildly elevated glutamine at 713 *μ*M (337–673) with an elevated alanine to lysine ratio of 4.4. Elevated whole blood lactate persisted until day of life 7 and normalized thereafter. An echocardiogram on day of life 4 revealed moderately diminished left ventricular systolic function, with an ejection fraction of 45.2% and mildly diminished right ventricular dysfunction.

Due to concerns for a mitochondrial disorder in the setting of lactic acidosis, seizures, and depressed cardiac function, the patient was started on biotin, thiamine, and riboflavin. Rapid whole exome sequencing, SNP array, and mitochondrial DNA testing were also sent.

EEG was performed on day of life 4, which demonstrated multifocal seizures. He was started on phenobarbital, Keppra, and caffeine. A brain ultrasound image on day of life 4 was unremarkable. Continuous EEG starting on day of life 5 showed burst suppression with epileptiform activity, and he was intubated for airway concerns in the setting of continued seizure activity. A brain MRI scan on day of life 5 revealed mild diffuse thinning of the corpus callosum. Due to ongoing seizures on day of life 6, he was started on fosphenytoin and midazolam. In the following days, the patient had resolution of his seizures, and he was weaned off phenytoin and midazolam and extubated on day of life 11. Repeat echocardiogram on day of life 5 was normal.

On day of life 12, whole exome sequencing results revealed a homozygous pathogenic variant in ALDH7A1: c.1279G > *C* (p.E427Q). The parents, who were a nonconsanguineous Caucasian couple, were each shown to be a carrier of this variant. The patient was started on triple therapy of pyridoxine 100 mg/day, as well as total arginine supplementation of 200 mg/kg/day and a lysine-free and low tryptophan formula (GA1 Anamix® Early Years, Nutricia) that was titrated to keep his plasma lysine levels in the lower limit of normal. Urine pipecolic acid that was sent prior to starting therapy was significantly elevated at 1687 *μ*mol/g creatinine (normal range <200 *μ*mol/g creatinine). After approximately 1 week of treatment, urine pipecolic acid normalized to 119 *μ*mol/g creatinine, providing further biochemical evidence of a diagnosis of PDE.

The infant did well after diagnosis and starting of triple therapy and was ultimately discharged home with no further seizures noted. At 3 months of age, he remained seizure free on his metabolic formula and was noted to be developing normally.

## 3. Discussion

In the present patient, who presented at 3 days of life with seizures, fever, respiratory distress, decreased cardiac function, and lactic acidosis, the differential diagnosis was initially wide and included mitochondrial disorders, infectious processes, inborn errors of metabolism, and other single-gene etiologies for infantile seizures. The diagnosis of PDE due to biallelic pathogenic variants in ALDH7A1 was made by 12 days of life via a rapid whole exome sequencing test.

The molecular diagnosis clarified the etiology of the seizure presentation, though some atypical features of this patient required further consideration. Although elevated blood lactate has been previously reported in patients with PDE, in the present patient, the elevated lactate level was likely exacerbated by ongoing seizure activity because lactate levels normalized as seizure activity resolved. The fever and respiratory distress may have reflected an infectious process that was not elucidated on cultures, though these symptoms resolved as the seizures resolved. The acute changes in left ventricular cardiac function are more difficult to explain, although cardiomyopathy was described in a prior infant with PDE [8]. In the present patient, the cardiac dysfunction resolved within 24 hours, and the clinical significance remains unknown.

In retrospect, a trial of pyridoxine early in the seizure presentation would have likely clarified the diagnosis, and we note that it is critical for institutional protocols to be in place in order to facilitate timely diagnoses of rare disorders. If this infant experienced cessation of seizures with antiepileptic drug therapy, a pyridoxine trial would not have revealed an apparent diagnosis. This highlights the fact that in cases of pyridoxine responsive epilepsy with an initial response to AEDs, diagnosis becomes more difficult. These patients often undergo multiple hospitalizations through the course of several years before a diagnosis is made and appropriate treatment is initiated.

Genetic testing, as in our patient, can be critical for establishing the diagnosis in an individual by identification of biallelic pathogenic variants in the gene ALDH7A1. Therapy for PDE consists of a three-part approach: pyridoxine supplementation, low lysine diet, and arginine supplementation [9]. This triple therapy has evolved from monotherapy with pyridoxine supplementation as we gain better understanding of the biomolecular pathways involved in lysine catabolism. Pyridoxine supplementation treats one of the most profound aspects of PDE-clinical and subclinical seizures noted on EEG in these patients. This does not, however, correct the underlying inability to catabolize lysine. Metabolite precursors in this pathway are thought to be neurotoxic, and administration of pyridoxine does not correct the accumulation of these potential toxins. Thus, a low lysine diet is administered in addition to pyridoxine supplementation.

A lysine-restricted diet has also been the mainstay treatment of glutaric aciduria type I (GA1), an inborn error of metabolism in which defects further down the lysine catabolism pathway cause damage via toxic levels of glutaric acid (GA), 3-hydroxy glutaric acid (3-OH-GA), and glutaconic acid. In addition to lysine restriction, individuals with GA1 are also treated with supplemental arginine. Arginine competes with lysine for transport across the blood-brain barrier and thus lowers levels of lysine transported into the brain [10]. This principle has been adapted to PDE treatment to further achieve lower levels of lysine in the brain as a method to decrease the accumulation of toxic metabolites in patients with PDE. Of note, GA1 Anamix is a supplement developed for GA1 and is not only lysine free but also low in tryptophan. In PDE, tryptophan restriction is not necessary and may require dietary supplementation based on plasma amino acid levels/monitoring.

Triple therapy shows promise in improving neurocognitive developemental outcomes in individuals with PDE [11–13]. However, long-term follow-up is necessary, as these changes to management have been relatively recent. Triple therapy has been shown to lower levels of plasma AASA and P6C and potential neurotoxic metabolites that may be useful in trending effectiveness of therapy. However, AASA and P6C are unstable at room temperature and must be processed and frozen at −80°C. Only few laboratories worldwide are equipped to run levels of AASA or P6C, and thus, PIP may be a more practical marker to test in treatment efficacy. Although PIP is a metabolite in the lysine catabolism pathway and a marker of PDE, it is not specific, as levels may be elevated in peroxisomal disorders. Additionally, it has not always been found to be elevated in patients with PDE and may be affected by administration of just pyridoxine alone [14]. However, in patients who are found to have high levels of PIP prior to treatment, it can be followed as a marker to trend for treatment efficacy as in our patient.

Diagnostic challenges of PDE include the rarity of the condition, as well as the frequent atypical clinical presentation. Genetic testing is especially valuable in patients who present with atypical PDE. In our patient, because seizures initially responded to multiple AEDs and comorbidities including cardiac dysfunction somewhat obscured the diagnosis, a pyridoxine trial was deferred. However, rapid WES was able to elucidate a diagnosis that in a timely manner allowing for initiation of therapy in a crucial period of development.

WES has become a powerful diagnostic tool in the ICU setting, largely due to advances in high throughput sequencing and analysis that have drastically reduced the turn-around time to a week. Particularly in cases involving rare conditions with variable or atypical presentations, diagnosis has historically been more challenging. There has been one other case report diagnosing an atypical presentation of PDE on WES, although in an outpatient setting [15]. This case highlights the utility of rapid WES in aiding in diagnosis and directly impacting management in an atypical presentation of PDE in the ICU setting, particularly as the initial concern was of a mitochondrial disorder. As rapid WES becomes more readily available and accessible, diagnoses that have historically remained elusive may occur earlier in the disease course having significant impacts on treatment and clinical outcomes.

## 4. Conclusion

In the neonate presenting with seizures unresponsive to AEDs and without obvious causes, a trial of pyridoxine should be performed as a first-line approach. This can be complemented by biochemical testing that supports a diagnosis for PDE (i.e., pipecolic acid), though this specialized testing is not always readily available. However, the diagnosis of atypical PDE has historically been challenging because some individuals do respond to AEDs and do not initially respond to pyridoxine. Additionally, atypical or confounding clinical comorbidities, as in our patient, may make a diagnosis unclear. Genetic testing may expedite diagnosis and treatment and serves as a critical diagnostic approach to complement therapeutic trials or biochemical testing, particularly in nonclassical cases. Early treatment using triple therapy from the recently published consensus guidelines achieves excellent control of seizures and may also prevent future neurocognitive delays if implemented early enough in the disease course. However, long-term follow-up studies of patients treated with triple therapy are necessary to determine the impact of improved lysine control on neurocognitive development.

## Data Availability

Data sharing is not applicable to this article as no new data were created or analyzed in this study.

## References

[1] Coughlin C. R., Swanson M. A., Spector E. (2019). The genotypic spectrum of ALDH7A1 mutations resulting in pyridoxine dependent epilepsy: a common epileptic encephalopathy. *Journal of Inherited Metabolic Disease*.

[2] Zuberi S. M., Wirrell E., Yozawitz E. (2022). ILAE classification and definition of epilepsy syndromes with onset in neonates and infants: position statement by the ILAE Task Force on Nosology and Definitions. *Epilepsia*.

[3] Hunt A. D., Stokes J., McCrory W. W., Stroud H. H. (1954). Pyridoxine dependency: report of a case of intractable convulsions in an infant controlled by pyridoxine. *Pediatrics*.

[4] Boehm T., Hubmann H., Petroczi K. (2020). Condensation of delta‐1‐piperideine‐6‐carboxylate with ortho‐aminobenzaldehyde allows its simple, fast, and inexpensive quantification in the urine of patients with antiquitin deficiency. *Journal of Inherited Metabolic Disease*.

[5] Mills P. B., Struys E., Jakobs C. (2006). Mutations in antiquitin in individuals with pyridoxine-dependent seizures. *Nature Medicine*.

[6] Osman C., Foulds N., Hunt D., Jade Edwards C., Prevett M. (2020). Diagnosis of pyridoxine-dependent epilepsy in an adult presenting with recurrent status epilepticus. *Epilepsia*.

[7] Bankier A., Turner M., Hopkins I. J. (1983). Pyridoxine dependent seizures—a wider clinical spectrum. *Archives of Disease in Childhood*.

[8] van Karnebeek C. D., Tiebout S. A., Niermeijer J. (2016). Pyridoxine-dependent epilepsy: an expanding clinical spectrum. *Pediatric Neurology*.

[9] Coughlin C. R., Tseng L. A., Abdenur J. E. (2021). Consensus guidelines for the diagnosis and management of pyridoxine-dependent epilepsy due to *α*-aminoadipic semialdehyde dehydrogenase deficiency. *Journal of Inherited Metabolic Disease*.

[10] Schmidt Z., Murthy G., Ennis M., Stockler-Ipsiroglu S., Elango R. (2020). Impact of enteral arginine supplementation on lysine metabolism in humans: a proof-of-concept for lysine-related inborn errors of metabolism. *Journal of Inherited Metabolic Disease*.

[11] Al Teneiji A., Bruun T. U. J., Cordeiro D. (2017). Phenotype, biochemical features, genotype and treatment outcome of pyridoxine-dependent epilepsy. *Metabolic Brain Disease*.

[12] Coughlin C. R., van Karnebeek C. D., Al-Hertani W. (2015). Triple therapy with pyridoxine, arginine supplementation and dietary lysine restriction in pyridoxine-dependent epilepsy: neurodevelopmental outcome. *Molecular Genetics and Metabolism*.

[13] Minet P., Sarret C., Miret A., Mention K., Benoist J. F., Remerand G. (2021). Clinical and biochemical outcome of a patient with pyridoxine-dependent epilepsy treated by triple therapy (pyridoxine supplementation, lysine-restricted diet, and arginine supplementation). *Acta Neurologica Belgica*.

[14] Xue J., Wang J., Gong P. (2019). Simultaneous quantification of alpha-aminoadipic semialdehyde, piperideine-6-carboxylate, pipecolic acid and alpha-aminoadipic acid in pyridoxine-dependent epilepsy. *Scientific Reports*.

[15] Haidar Z., Jalkh N., Corbani S., Fawaz A., Chouery E., Mégarbané A. (2018). Atypical pyridoxine dependent epilepsy resulting from a new homozygous missense mutation, in ALDH7A1. *Seizure*.

